# Modeling lottery incentives for daily adherence

**DOI:** 10.1002/sim.8149

**Published:** 2019-04-02

**Authors:** Colman H. Humphrey, Dylan S. Small, Shane T. Jensen, Kevin G. Volpp, David A. Asch, Jingsan Zhu, Andrea B. Troxel

**Affiliations:** ^1^ Wharton School University of Pennsylvania Philadelphia Pennsylvania; ^2^ Perelman School of Medicine University of Pennsylvania Philadelphia Pennsylvania; ^3^ LDI Center for Health Incentives and Behavioral Economics University of Pennsylvania Philadelphia Pennsylvania; ^4^ Center for Health Equity Research and Promotion Crescenz Veterans Affairs Medical Center Philadelphia Pennsylvania; ^5^ The Penn Medicine Center for Health Care Innovation University of Pennsylvania Philadelphia Pennsylvania; ^6^ Department of Population Health New York University School of Medicine New York New York

**Keywords:** autocorrelated, binary, incentive, interrupted time series, lottery, quasi‐experiment

## Abstract

Many health issues require adherence to recommended daily activities, such as taking medication to manage a chronic condition, walking a certain distance to promote weight loss, or measuring weights to assess fluid balance in heart failure. The cost of nonadherence can be high, with respect to both individual health outcomes and the healthcare system. Incentivizing adherence to daily activities can promote better health in patients and populations and potentially provide long‐term cost savings.

Multiple incentive structures are possible. We focus here on a daily lottery incentive in which payment occurs when both the participant's lottery number matches the number drawn and the participant adheres to the targeted daily behavior.

Our objective is to model the lottery's effect on participants' probability to complete the targeted task, particularly over the short term. We combine two procedures for analyzing such binary time series: a parameter‐driven regression model with an autocorrelated latent process and a comparative interrupted time series. We use the output of the regression model as the control generator for the comparative time series in order to create a quasi‐experimental design.

## 1 MODELING LOTTERY INCENTIVES: AN INTRODUCTION

### 1.1 Adherence to daily activities

Many chronic health issues require daily (or multiple times daily) adherence to medication for optimal management; examples include diabetes, hypertension, and hypercholesterolemia. Some conditions, such as obesity, can be improved with daily physical activity. Other issues, such as addiction or substance abuse, are treated with abstinence programs in which the daily goal is nonuse of the substance.

Many of these health‐promoting activities can be defined as adherence to a recommended daily task. If medications are to be taken once or more per day, adherence constitutes taking all of the required pills for the day; failure to take any portion would constitute nonadherence. Adherence to a daily activity can be defined as reaching a specified target, such as 7000 steps walked per day. In abstinence studies, adherence can be defined as achievement of an abstinent day.

The purpose of defining health behaviors as adherence to a daily task is twofold: it permits both monitoring of activity and incentivizing such activity. While adherence can be defined for any period, longer periods make it difficult to decipher short‐term behavior effects and make individual daily behaviors less salient. On the other hand, too‐frequent monitoring (eg, twice‐daily pill taking) may require burdensome evaluation and feedback, causing a disassociation between adherence and intervention.

### 1.2 Incentive mechanisms

There are a variety of ways to structure financial incentives, including as fixed payments, daily lotteries, pre‐commitment devices such as deposit contracts, or with nonmonetary rewards. We focus here on daily lotteries in which “winning” is conditional upon fulfillment of the targeted daily activity. These lotteries incorporate several powerful concepts derived from behavioral economics, a field that incorporates both economic principles and insights from psychology to encourage good decision‐making and effect positive behavior change. For example, we tend to misinterpret small probabilities, a phenomenon that may explain the popularity of state lotteries with very low expected values. In addition, we experience “loss aversion,” in which the loss of a certain size is more distressing than a gain of equivalent size is reinforcing.^1^ We also experience “regret aversion,” in which the emotional cost of regret (eg, having missed the chance at a reward) is significant.^2^ Using these concepts, we have designed “regret lotteries” that take advantage of many of these concepts to encourage desired behavior. Our group has conducted many trials of various lottery incentives for daily behaviors in an effort to improve health behaviors in a variety of contexts; two examples regarding medication adherence are given in the following.

In this paper, we aim to model the lottery program's effect on adherence to a daily medication regime. We model daily adherence, as a function of the daily lottery outcomes. Our main goal is to understand the mechanism of the lottery, and how it affects both short‐ and longer‐term adherence. We wish to form hypotheses about future lottery incentive structures, including how to best allocate a fixed amount of money. 1More specifically, a fixed expected amount per adherent day: the lottery naturally adds a random element to payouts, and in all payment structures, payouts grow linearly with adherence, as is desired.


### 1.3 Binary time series

The analysis of autocorrelated time series with binary outcomes is less straightforward than analysis for the continuous equivalent, as we cannot apply well‐developed Gaussian methods. In place of autoregressive integrated moving average models, binary models can use generalized linear autoregressive integrated moving average models; these models are referred to as observation‐driven, because the distribution of the outcome at a given time *t* depends explicitly on prior observations, and not on a hidden process.

In contrast to observation‐driven methods, “parameter‐driven” methods incorporate a latent process to account for dependence. Kalman filtering is an example in continuous settings,^3^ and more generally, we can apply hidden Markov models^4^ and dynamic Bayesian networks.^5^ It is common to assume a discrete hidden structure to underlie a discrete time series, but for many applications, including this paper's application, discrete hidden states do not offer much inferential benefit over observation‐driven methods. Similar to the work of Wu and Cui,^6^ we will assume a continuous underlying process.

The resulting model has useful inferential properties in its own right: we obtain information about both the underlying autoregressive structure and the directionality and significance of our covariates. Following the work of Campbell and Stanley,^7^ we then analyze our multiple time series as comparative interrupted time series, using the output of our regression models as the control mechanism for comparative time series.

## 2 LOTTERY STRUCTURE

In our trials, lottery incentive group members were first asked to choose a personal two‐digit number between 00 and 99. Every day, a random number was selected as the winning lottery number. If a participant's number matched the lottery on one digit (18% chance), s/he was eligible to win a small amount; if the participant's number matched both digits (1% chance) s/he was eligible to win a large amount. The “win” amounts varied slightly by trial; the small prize was $5 or $10, and the large prize was $50 or $100, resulting in expected values of approximately $1.40 or $2.80, respectively. An important feature of the lottery is that the participant received their winnings *only if s/he had been adherent the previous day*. This “regret” feature, along with the variable reinforcement produced by randomness in the frequency of winning and variation in the magnitude of the prize, was designed to enhance the motivational strength of the lottery.

We present analyses of two different studies using lottery incentive interventions to motivate daily behaviors, described in the following. Both studies provided financial incentives for patients to take medications that they were already being prescribed, and were reviewed and approved by the Institutional Review Board at the University of Pennsylvania.

### 2.1 Medication adherence and hyperlipidemia

The Shared Incentives study^8^ was designed to incentivize adherence to cholesterol‐lowering medication (ie, statins), and included four treatment arms: a control group, a physician incentive group (physicians received direct payments), a participant incentive group (participants entered in a daily lottery like that described above), and a shared physician and participant incentive group (both received incentives at half value). To demonstrate our approach, we focus on the participant incentive groups here.

Both groups receiving participant incentives participated in a lottery as detailed in the previous section, with the participant incentive group receiving $100 and $10 for large and small wins, respectively, and the Shared Incentive group receiving $50 and $5. The trial enrolled 1503 participants, and followed them for 1 year of intervention, and an additional 3 months following the intervention; attrition was less than 10%. Participants in the control group and physician incentive group were not part of the lottery intervention; we therefore combine both groups and consider them the control group here.

### 2.2 Medication adherence after myocardial infarction

The HeartStrong study,^9^, ^10^ designed to incentivize adherence to beneficial medications following a heart attack (ie, statins, aspirin, beta‐blockers, and anti‐platelet medications), included a control and an incentive arm. The incentive arm received the same lottery as detailed above, with large win amounts of $50 and small win amounts of $5, along with social support from a personal supporter and study‐supported social worker. The trial enrolled 1509 participants, and followed them for 1 year of intervention, and an additional 3 months following the intervention; attrition was less than 10%. The control group received usual care with no intervention.

## 3 DATA DESCRIPTION AND ESTIMATES OF INTEREST

Our general problem consists of participants *i* = 1,…,*N*, each with a time series ${\left\{Y_{t}\right\}}^{i}$ over a set study period *t* = 1,…,*T*_*i*_ (due to start‐up issues with study devices, *T*_*i*_ is often participant‐dependent), with *Y*_*i*,*t*_ = 1 if the goal is completed, and 0 otherwise. Most of our studies are of the form

$$Y_{i,t}=\left\{\begin{array}{cc} 1, & \text{pill taken by participant}\;i\;\text{on day}\;t\\ 0, & \text{otherwise}, \end{array}\right.$$

or

$$Y_{i,t}=\left\{\begin{array}{cc} 1, & \text{participant}\;i\;\text{walks}\geq 7000\;\text{steps on day}\;t\\ 0, & \text{otherwise}. \end{array}\right.$$

For the case of medication adherence, a binary time series is the natural choice.

If the goal is completed on day *t* and the participant has matching number(s), then the participant is awarded the lottery winnings and is informed. If the goal is not completed but the participant has matching number(s), the participant receives a “regret” message telling her/him that s/he *would have* won, if s/he had only completed the goal. We represent these outcomes with four indicators, with *l* and *L* referring to the small and large lotteries, respectively, and *w* and *r* referring to wins and regrets:

(1) $$\begin{array}{ccc} l_{w} & =\left\{\begin{array}{cc} 1, & \text{small win},Y=1\\ 0, & \text{otherwise} \end{array}\right.\;l_{r}=\left\{\begin{array}{cc} 1, & \text{small win},Y=0\\ 0, & \text{otherwise} \end{array}\right. & \\ L_{w} & =\left\{\begin{array}{cc} 1, & \text{large win},Y=1\\ 0, & \text{otherwise} \end{array}\right.\;L_{r}=\left\{\begin{array}{cc} 1, & \text{large win},Y=0,\\ 0, & \text{otherwise}. \end{array}\right. \end{array}$$

These are participant‐ and day‐dependent, so we collect them to give $L_{t}^{i}={\left(l_{w},l_{r},L_{w},L_{r}\right)}_{t}^{i}$. Note that, at most, one element of $L_{t}^{i}$ can take the value 1, and that most of the time (approximately 81% in the studies described here), $L_{t}^{i}=(0,0,0,0)$, ie, no lottery winnings occurred.

The lottery results for participant *i* on day *t* are given after *Y*_*i*,*t*_ is recorded; thus, our covariates for day *t* include a function only of $\left\{L_{1}^{i},\text{…},L_{t-1}^{i}\right\}$, lottery results from days prior to *t*.

### 3.1 Lottery assessment: full study effects

We can define the ATE, as in Equation (2) (ATE during the study period) and Equation (3) (ATE during the follow‐up period) in the following, to describe the total value of the lottery:

(2) $${\text{ATE}}_{\text{in‐study}}=E\left[\overset{T_{i}}{\underset{t=1}{\sum }}Y_{i,t}^{\text{participant}\;i\;\text{in lotto arm}}\right]-E\left[\overset{T_{i}}{\underset{t=1}{\sum }}Y_{i,t}^{\text{participant}\;i\;\text{in control arm}}\right]$$

(3) $${\text{ATE}}_{\text{post‐study}}=E\left[\underset{t>T_{i}}{\sum }Y_{i,t}^{\text{participant}\;i\;\text{in lotto arm}}\right]-E\left[\underset{t>T_{i}}{\sum }Y_{i,t}^{\text{participant}\;i\;\text{in control arm}}\right].$$

These values, however, are not our main objective; they are more properly compared with aggregation methods used in, eg, the works of Patel et al^11^ and Troxel et al.^9^ Our goal is instead to analyze short‐term response to the lottery, in order to understand the mechanism of the lottery's effect and thus inform the optimal design for future lotteries.

It is possible that the entire effect of the lottery mechanism is nonresponsive to messaging: that is, participants adjust their baseline goal‐completion rate due to the knowledge of being in the lottery arm, and conditional on this have no response to daily messaging and payouts. It is also possible that the lottery could be beneficial in terms of the ATE but produce a locally negative effect. For example, winning the lottery could make participants decide that they have earned a day off, leading them to be nonadherent the following day. If this is the case, then prior psychological knowledge, or running many lottery experiments of different types, would best inform lottery design. We will not be totally left in the dark in such a scenario, as we can compare local, or short‐term, effects of the lottery with the ATE from aggregated models. Patterns found under such a comparison can partially inform lottery design.

### 3.2 Lottery assessment: short‐term effects

We would like to determine the effect over *d* days of winning the various lotteries on day *τ* for a given participant. Using TE for the treatment effect, this would be

(4) $$\text{TE}(\text{small lottery},d,\tau ,i)=\left[\overset{\tau +d}{\underset{t=\tau +1}{\sum }}Y_{i,t}^{\text{participant}\;i\;\text{wins small lottery on day}\;\tau }\right]-\left[\overset{\tau +d}{\underset{t=\tau +1}{\sum }}Y_{i,t}^{\text{participant}\;i\;\text{loses small lottery on day}\;\tau }\right],$$

where winning the small lottery requires a success on day *τ*, ie, $Y_{\tau }^{i}=1$, and similarly, losing requires $Y_{\tau }^{i}=1$. The equivalent for the regret lotteries would require failure, ie, $Y_{\tau }^{i}=0$ in both the treated and control units.

Using our notation from earlier with lottery results, we could write the above equation

(5) $$\text{TE}(l_{w},d,\tau ,i)=\left[\overset{\tau +d}{\underset{t=\tau +1}{\sum }}Y_{i,t}^{\left\{{\left(l_{w}\right)}_{\tau }^{i}=1,Y_{\tau }^{i}=1\right\}}\right]-\left[\overset{\tau +d}{\underset{t=\tau +1}{\sum }}Y_{i,t}^{\left\{{\left(l_{w}\right)}_{\tau }^{i}=0,Y_{\tau }^{i}=1\right\}}\right].$$

Because we cannot observe counterfactuals, we instead compute the expected value of this quantity, using all participants

(6) $$\text{ATE}(l_{w},d,\tau )=E\left[\overset{\tau +d}{\underset{t=\tau +1}{\sum }}Y_{i,t}^{\left\{{\left(l_{w}\right)}_{\tau }^{i}=1,Y_{\tau }^{i}=1\right\}}\right]-E\left[\overset{\tau +d}{\underset{t=\tau +1}{\sum }}Y_{i,t}^{\left\{{\left(l_{w}\right)}_{\tau }^{i}=0,Y_{\tau }^{i}=1\right\}}\right].$$

In general, computing these quantities separately for each *τ* is very noisy. We can define the full study local lottery effect for *d* days as

(7) $$\text{ATE}(l_{w},d,\text{Full study})=E_{\tau }\;\text{ATE}(l_{w},d,\tau ).$$

Similarly, we have ATE(*L*_*w*_,*d*,Full study), ATE(*l*_*r*_,*d*,Full study), and ATE(*L*_*r*_,*d*,Full study). The number of days *d* can be chosen by the practitioner; we use *d* ∈ {1,5,10}.

## 4 MATCHING AND MODELING DAILY ADHERENCE

In this section, we outline our two‐pronged approach for our matching analysis. Our main interest is to compare the difference in adherence between lottery winners and nonwinners, and similarly, between those who received a regret message and those who did not. Once the controls, ie, the appropriate comparison group, are chosen, this method is straightforward.

Choosing suitable controls is the most difficult aspect of most matching analyses, and the same is true here. We want to match people with an approximately similar base rate of adherence at the time of the comparison, or else our differences in adherence rates will not correctly estimate the effect of the lotteries. To this end, we cannot match people based on overall adherence: this is partly due to post‐treatment matching bias,^12^ but also ignores the variance in adherence probability for each participant over the course of a study.

We propose to model the daily adherence probability for each participant. We use the output of this modeling procedure to generate controls for our matching procedure. We could also match on recent adherence; we compare this method to ours in Section 7.4.

### 4.1 Data structure and latent processes

We have *N* time series, ${\left\{Y_{t}\right\}}^{i}$, *i* ∈ {1,…,*N*}. Each time series is a binary sequence, *Y*_*i*,*t*_ ∈ {0,1}, *t* = 1,…,*T*_*i*_, corresponding to daily adherence. Each sequence has a sequence of associated covariates, ${\left\{U_{t}\right\}}^{i}$, and a parameter vector ***β*** ^*i*^. The covariate vector includes a function of $\left\{L_{1}^{i},\text{…},L_{t-1}^{i}\right\}$, ie, all lottery results up to day (*t* − 1). 2The lottery on day *t* is a function of *Y*_*t*_; thus, $L_{t}$ cannot be a predictor for *Y*_*t*_. The covariate vector ${\left\{U_{t}\right\}}^{i}$ could also include other clinically relevant variable such as participant demographics or baseline characteristics.

A standard generalized linear model assumes that the mean of *Y* is a function *G*(.) of ${\left(\beta^{i}\right)}^{\prime }U_{t}^{i}$, where *G*(.) is a function from R→(0,1), typically, a CDF such as the logistic function or the Gaussian CDF. The issue is the lack of independence: the unconditional mean $E[Y_{i,t}\;|\;U_{t}^{i}]$ is unlikely to be the same as the conditional mean $E[Y_{i,t}\;|\;Y_{t-1}^{i},U_{t}^{i}]$. Note that, for binary data, **E**(*Y*) = **P**(*Y* = 1).

We can solve the correlation issue in multiple ways. From the work of Cox et al,^13^ the two most general descriptions are observation‐driven models and parameter‐driven models. In observation‐driven models, *Y*_*i*,*t*_ depends explicitly on prior values $Y_{\tau }^{i}$ for *τ* < *t*; see the GLARMA package^14^ for R. In parameter‐driven models, we assume a hidden state on which *Y* depends. We believe that parameter‐driven models offer a more natural interpretation of the process of the time series. Under simulation, they project less bias onto future predictions. The disadvantage is in fitting these models.

We let $X_{t}^{i}$ represent this hidden state, or underlying process. That is, for each binary sequence ${\left\{Y_{t}\right\}}^{i}$, there is a corresponding continuous sequence ${\left\{X_{t}\right\}}^{i}$, on which the binary sequence depends. Specifically, we assume that

$$\mathrm{Pr}(Y_{i,t}=1\;|\;X_{t})=\Phi \left(X_{t}^{i}\right),$$

where Φ is the normal CDF.

We assume that $X_{t}^{i}$ directly incorporates the unit level covariates, $U_{t}^{i}$. Further, we assume an autocorrelation parameter *φ* ^*i*^ ∈ ( −1,1) on the error structure for the underlying process

(8) $$\left\{\begin{array}{c} X_{t+1}^{i}={\left(\beta^{i}\right)}^{\prime }U_{t+1}^{i}+\eta_{t+1}^{i}\\ \eta_{t+1}^{i}\;=\varphi^{i}\eta_{t}^{i}+\varepsilon_{t+1}^{i}. \end{array}\right.$$

Combining this in one step, we have

(9) $$X_{t+1}^{i}-{\left(\beta^{i}\right)}^{\prime }U_{t+1}^{i}=\varphi^{i}\left(X_{t}^{i}-{\left(\beta^{i}\right)}^{\prime }U_{t}^{i}\right)+\varepsilon_{t+1}^{i},$$

with $\varepsilon_{t+1}^{i}$ being a zero mean, IID variable such that $\mathrm{Var}(\varepsilon_{t+1}^{i})=\sigma_{i}^{2}$. Unconditionally, Equations (8) give $E(X_{t}^{i})={\left(\beta^{i}\right)}^{\prime }U_{t}^{i}$. It generally will not be necessary to assume that *ε* is normally distributed.

This model is equivalent to having an underlying process *α*_*t*_, with $P(Y_{t}\;|\;U_{t})=\Phi (\beta^{\prime }U_{t}+\alpha_{t})$, with *α*_*t*_ being an autoregressive mean zero process with no covariates.

We further discuss what our unit level covariates $U_{t}^{i}$ contain in the next section. Note that our notation differs from typical regression models, in that $U_{t}^{i}$, and not $X_{t}^{i}$, would contain clinical variables of importance if they were to be included in the model.

### 4.2 Structure of U and decaying lottery effect

Our predictors, the $U_{t}^{i}$ vectors, contain an intercept, a time variable, and prior lottery results. The time variable is to account for potentially changing base goal completion rates over the course of the study.

As detailed in our data description and Equations (1), we allow the lottery to affect the underlying processes ${\left\{X_{t}\right\}}^{i}$ in four ways: when you win the lottery, and when you would have won if you had been eligible, ie, when you receive a regret message; and for each, when the amount is large, and when the amount is small. We called this collection of mutually exclusive indicators $L_{t}^{i}$, with $L_{t}^{i}={\left(l_{w},l_{r},L_{w},L_{r}\right)}_{t}^{i}$.

Further, we allow these lottery effects to propagate beyond the next day, into the future. If the lottery only affected the next day, we would have

(10) $$U_{t}^{i}=\left[1,\;t,\;{\left(l_{w},l_{r},L_{w},L_{r}\right)}_{t-1}^{i}\right].$$

Instead, we allow ***U*** to contain a function of prior lottery results. The method is detailed in the following section. Essentially ***U*** contains a power decayed function of the most recent lottery, with the rates of decay also parameters in our model. Each lottery effect is assumed to be strongest initially, but may continue to have some effect for future days. Our models allow both the shape of the decay and the length of the decay to vary. We fit the decay parameters separately for the large and small lotteries.

### 4.3 Decaying lottery effect

In allowing the lottery to affect future days, we set up a decaying structure on all four lottery effects. The data do not, in general, have enough signal to strongly identify separate decay parameters for winning and regret, so we fit the same parameters for the two large effects, large wins and large regrets, and a separate set of parameters for the small effects, small wins and small regrets.

We assume decay is parameterized by (*γ*,*λ*), ie, we have some function *G*(*x*,*γ*,*λ*) that gives the weight of values from *x* days ago. We assume that *G*(0,*γ*,*λ*) = 1 for all *γ* and *λ*, so that all decay is relative to day one.

*γ* can be thought of as the shape parameter, and *λ* the length parameter. *λ* is how many days the lottery lasts for, so that *G*(*x*,*γ*,*λ*) = 0 for any *x* > *λ*. *γ* controls how the effect scales down to zero at *x* = *λ*: when *γ* = 1, the decay is linear; for *γ* < 1, the effect decays more rapidly than linearly, and for *γ* > 1, the values decays more slowly than linearly, ie, the effect of the lottery is stronger for longer.

For these applications, we use the following functional form:

(11) G(x,γ,λ)=1−(xλ)γγx≤λ0x>λ.

While *x* will only ever be an integer in our examples, this function is defined for all *x* ≥ 0. *λ* can also be any real number, not just an integer.

We can then use the propagated lottery effects in ***U***. Note that we propagate only the most recent effect. Recall our vector of lottery effects, $L_{t}^{i}={\left(l_{w},l_{r},L_{w},L_{r}\right)}_{t}^{i}$. Without loss of generality, we focus here on just one effect, eg, the small wins, *l*_*w*_, on day *t* for person *i*.

If one of the other three results happened more recently, that is, if there was a large win, a large regret, or a small regret more recently than a small win, we set ${\left(l_{w}\right)}_{t}^{i}=0$. If a small win was the most recent result, assume that occurred *d* days ago, with *d* ≥ 0. We set

$${\left(l_{w}\right)}_{t}^{i}=G(d,\gamma_{\text{small}},l_{\text{small}})\times {\left(l_{w}\right)}_{t-d}^{i}.$$

Of course, if *d* = 0, ie, the win occurred on day *t*, we set ${\left(l_{w}\right)}_{t}^{i}=1$. Thus, we can just eliminate the indicator from the above definition, to get

$${\left(l_{w}\right)}_{t}^{i}=\left\{\begin{array}{cc} G(d,\gamma_{\text{small}},l_{\text{small}}), & \text{won}\;d\;\text{days ago}\\ 0, & \text{other lottery result since}. \end{array}\right.$$

If no lottery result has happened at all yet, we set $L_{t}^{i}=(0,0,0,0)$.

**Figure 1:**
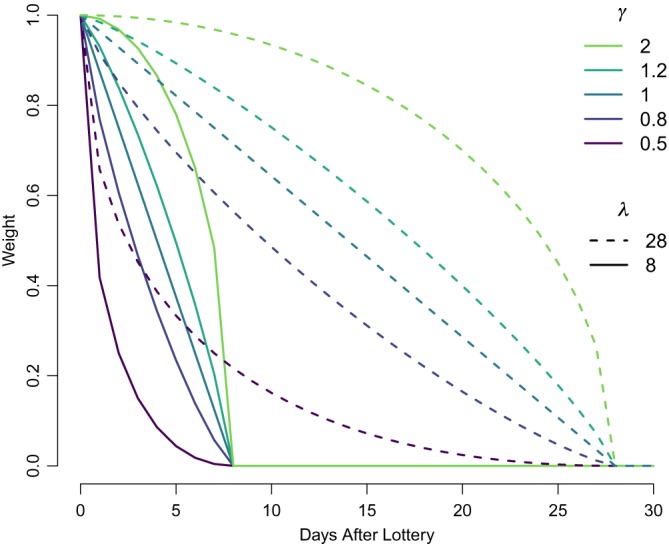
Decay curves for different γ and λ values [Colour figure can be viewed at wileyonlinelibrary.com]

For the small lotteries (win and regret), recall that we have *p*_small lotto_ = 0.18, and for the large, we have *p*_large lotto_ = 0.01. We assume a finite effect, with no effect after at most 10 days for the small lotteries, and 100 days for the large; ie, we limit *λ*_small_ to be less than 12, and *λ*_large_ to be less than 100. This allows a flexible decay pattern, as seen in Figure 1, which presents decay curves for varying values of *γ* and *λ*.

While we can have individual small lottery coefficients for participants, due to the large amount of noise in the data, we assume a shared value of *γ*_small_ and *λ*_small_ over all participants and, similarly, a shared *γ*_large_ and *λ*_large_.

### 4.4 Modeling methods for regression

If primary interest is inference for the ***β*** ^*i*^ vectors, we can follow the work of Wu and Cui^6^ or the work of Dunsmuir and He^15^ in marginalizing out the $\left\{X_{t}^{i}\right\}$ processes to get valid inference on ***β*** ^*i*^.

If we have inferential interest in *φ* ^*i*^ and the $\left\{X_{t}^{i}\right\}$ sequences themselves, we cannot aggregate through *X*, and must either solve a very high dimensional likelihood problem, or use Bayesian methods, similar to the work of Klingenberg.^16^ Bayesian methods also allow us to place a flexible hierarchical structure on our parameters. We can also make our problem more general and allow the parameter vectors to vary over time.

Details of our hierarchical setup are given in Appendix B.

### 4.5 Comparative time series

In theory, if our model is well fit and correct, we can solve integrals, or even simulate, to work out the unconditional effects of $U_{t}^{i}$ on future observations, $Y_{\tau }^{i}$, *τ* ≥ *t*. However, this requires our model to be well specified to obtain unbiased estimates and also requires our model to be fully identified. We would like to be able to make valid inference on the unconditional effects even if the best our model can do is have good predictive properties. If our model were misspecified and could only guarantee unbiasedness of the ${\left\{X_{t}\right\}}^{i}$ sequences, we would still like to obtain marginal inference. Further, as we will discuss in Section 4.7, the regression does not directly measure the lottery effects on adherence.

Assume we have a dichotomous covariate, $V_{\tau }^{i}$, which can be an element of our covariate vector $U_{\tau^{\prime }}^{i}$ at some potentially different time *τ* *′*. Assume it takes values in {0,1}. We can think of this as a treatment variable.

Our desire is to create quasi‐experimental data, following the work of Campbell and Stanley.^7^ For any given time *τ*, we can run one of two separate procedures: (a) run our model up to time *τ*; (b) take the results from our model run on the full dataset at time *τ*. From either of these two procedures, we will get a distribution for $X_{\tau }^{i}$ and, thus, a distribution for $P(Y_{\tau }^{i}=1)$.

We can separate our sequences into those with $V_{\tau }^{i}=1$ and those with $V_{\tau }^{i}=0$, and call these “treated” and “control,” respectively. We use the model output $X_{\tau }^{i}$ values to match “treated” sequences with “control” sequences. In our data, $V_{\tau }^{i}$ is the lottery result on day *τ* − 1. To be specific, it is an indicator variable for one of the four possible lottery results. Thus, we also match sequences on $Y_{\tau -1}^{i}$, to make sure *V* is just the effect of the lottery. That is, when two people are both eligible (ie, both adherent) but only one wins, we can match them on lotto wins; when two people are both not eligible (ie, both not adherent) and one gets a regret message, we can match them; when one person is eligible and the other is not, we cannot match them.

From here, we can apply the logic of interrupted time series: compare the subsequent sequences *Y*_*i*,*t*_, *t* ≥ *τ*, for the treated and control units. Because our sequences were matched at time *τ*, we do not have to fit time series models to the sequences: we can simply compare the differences and conclude the marginal effect of *V*. This gains us both internal and external validity, since we need not worry about the effects of *V* potentially being different for different *X* levels. The downside is that we can no longer use just any control subject for any treated subject. This leads us to the estimands in Section 3.2, ie, Equation (7): the average short‐term effect of each lottery type.

If *V* is continuous, we can still do this analysis, but we would have to change the strict definition of treated and control. One option would be to treat it as a dose‐response model.

### 4.6 Matching details

Here, we provide more concrete detail for the above section. For every day *t* of the study, we find our set of “treated” participants for each lottery effect. That is, we find all those who won the small lottery on day *t*, all those who had a small regret on day *t*, all who had a large win on day *t*, and all who had a large regret on day *t*. Our set of potential controls includes those who did not have a lottery effect on day *t*. To measure the effect of wins, we compare lottery winners only to participants who also completed the goal on day *t*, but did not win the lottery. To measure the effect of regret messages, we compare regret message receivers only to participants who also did not complete the goal on day *t* and thus were not eligible.

We have the option to match with or without replacement, ie, we can match each control to multiple treated subjects, or match each control to at most one treated subject. Allowing matching with replacement reduces bias because we can choose the closest possible controls for each treated subject.^17^ The downside is a potential increase in variance; however, in our case, blocking multiple uses of controls reduces our sample size enough to more than offset any variance decrease, so we match with replacement.

We determine the distance of each control to each treated participant based on **P**(*Y*_*t*_ = 1) = Φ(*X*_*t*_), the predicted probability of adherence on day *t*. We then compare $\Phi (X_{t}^{\text{control}})$ and $\Phi (X_{t}^{\text{treated}})$, and if they are within a given tolerance *δ*, the control participant is matched to that treated participant. We also allow many‐to‐one matching: multiple controls are used if available. In typical MCMC fashion, we compute Φ(*X*) for every iteration (post burn in), and average through them.

We make comparisons over 10 days, ie, we record the difference in adherence between our treated and control participants over a 10‐day period. Optionally, we can further restrict matches by using a cooling‐off period: a matched participant must wait a given number of days to be used in another comparison. If we use 5 days as a cooling‐off period, a participant cannot be part of a match on day 5 and also day 7—we will wait until day 11 to rematch him/her.

These matches give us a set of comparisons for all lottery types across the timeframe of the study. However, both of the choices above—the tolerance *δ* and the cooling‐off period—present trade‐offs in terms of the quality of each match and the total number of matches, ie, a classic bias‐variance trade‐off. We are not guaranteed to be able to match every treated subject at every time period. We discuss the implications of this further in Section 7.

One downside of matching with replacement is that the variance calculation becomes more difficult, particularly in many‐to‐one matching. We overcome this by bootstrapping to obtain the variance instead. Details are given in Appendix C.

### 4.7 Comparing regression with matching output

One might ask why we would want matched estimates after fitting our regression model. The primary reason is that the matched estimates more directly answer the question of interest: how much effect does the lottery have, and what is the mechanism? The direct estimates of the lottery effects also come with directly estimated standard errors.

The output of the regression method does not automatically inform us about the output of the matching method. For example, if a study has an extremely high adherence rate, this will limit the maximum effect size of the lottery: it is difficult to achieve a discernible positive difference in a participant's adherence rate if s/he is already completing the goal 98% of the time. The autoregressive parameters also affect the propagation of the lottery effects: a participant who nearly always just repeats today what s/he did yesterday will on average respond less strongly to any incentives. Thus, if we want to know the change in adherence from the lottery, we need to measure the change in adherence from the lottery.

On the other hand, the regression method does not suffer from these shortcomings; thus, we might expect our parameters to generalize beyond the scope of the study group more readily than the in‐sample lottery estimates.

## 5 MATCHING EXAMPLE AND GRAPHICAL SUMMARIES

Given the set of matches generated according to Section 4.6, we obtain a set of differences for all four lottery types. For each type, we may have multiple differences on a given study day, or none. Note that a given difference refers to one treated participant, and at least one but potentially many controls.

### 5.1 Matching example

Consider a specific example of the adherence vectors for the three units given in Table 1. If participant 45 wins the small lottery on day 13, we search for controls. Say that participant 45 has an expected value of *P*(*Y*) of 0.6 (corresponding to *X* = 0.25), 3We have **P**(*Y* = 1) = Φ(*X*), but indeed, **EP**(*Y* = 1) = **E**Φ(*X*) ≠ Φ(**E** *X*). We can match on **E** *X* instead of **E**Φ(*X*) if we want, but note that it will not be equivalent, and it is generally worse in simulation. and participants 21 and 62 both complete the goal on day 13 but do not win the lottery, and have expected values of *P*(*Y*) of 0.55 and 0.63, within our set threshold *δ* = 0.05, ie, |0.6 − 0.55| ≤ 0.05 and, similarly, |0.6 − 0.63| ≤ 0.05. We can therefore match these two participants with participant 45 on day 13. 4Note that it is possible that there might be no matches for participant 45 on this day. This could happen if no else completed the task that day; it could happen if everyone who completed the task also won the lottery; it could happen if no one eligible was within the required *δ* = 0.05 of 0.6; finally, it could happen if everyone eligible was in a cooling‐off period.


**Table 1:** Adherence after matching on day 13

	Study Day									
	14	15	16	17	18	19	20	21	22	23
45	1	0	0	1	1	0	0	1	1	0
21	1	1	1	0	1	0	0	0	0	0
62	0	1	0	0	0	0	1	1	0	0

With just one control, we simply subtract; with multiple controls we average the controls, then subtract. Thus, here, our difference vector would be

$${\text{Y}}_{14:23}^{45}-\left(\frac{{\text{Y}}_{14:23}^{21}+{\text{Y}}_{14:23}^{62}}{2}\right)=(0.5,-1,-0.5,1,0.5,0,-0.5,0.5,1,0).$$

Based on this comparison alone, our estimate for the 1‐day effect of the lottery would be an increase of 0.5; our 5‐day estimate would be 0.5 − 1 − 0.5 + 1 + 0.5 = 0.5, our 10‐day estimate would be the sum of all 10, or 1.

Of course we do not just use one comparison; hence, our estimate for the 1‐day effect is the average of the first element of all such difference vectors, the 5‐day estimate is the average of the sum of the first five element of all such vectors, and similarly for the 10‐day effect.

### 5.2 Graphical summaries

In the previous section, we provided an example of how we form the difference vectors for the matching process. We can aggregate these vectors in many ways; for example, we could stratify by different values of *X* to see the effect of the lotteries separately for high‐adherence and low‐adherence participants.

We produce an overall estimate of the effect of the four lotteries, for 1‐, 5‐, and 10‐day periods. Finally, we split the study in two with respect to time duration and compute the estimates separately for the first half and second half, to evaluate any estimated change in the effect of the lottery. These estimates are given with valid (bootstrapped) confidence intervals.

### 5.3 Shared Incentives results

**Figure 2:**
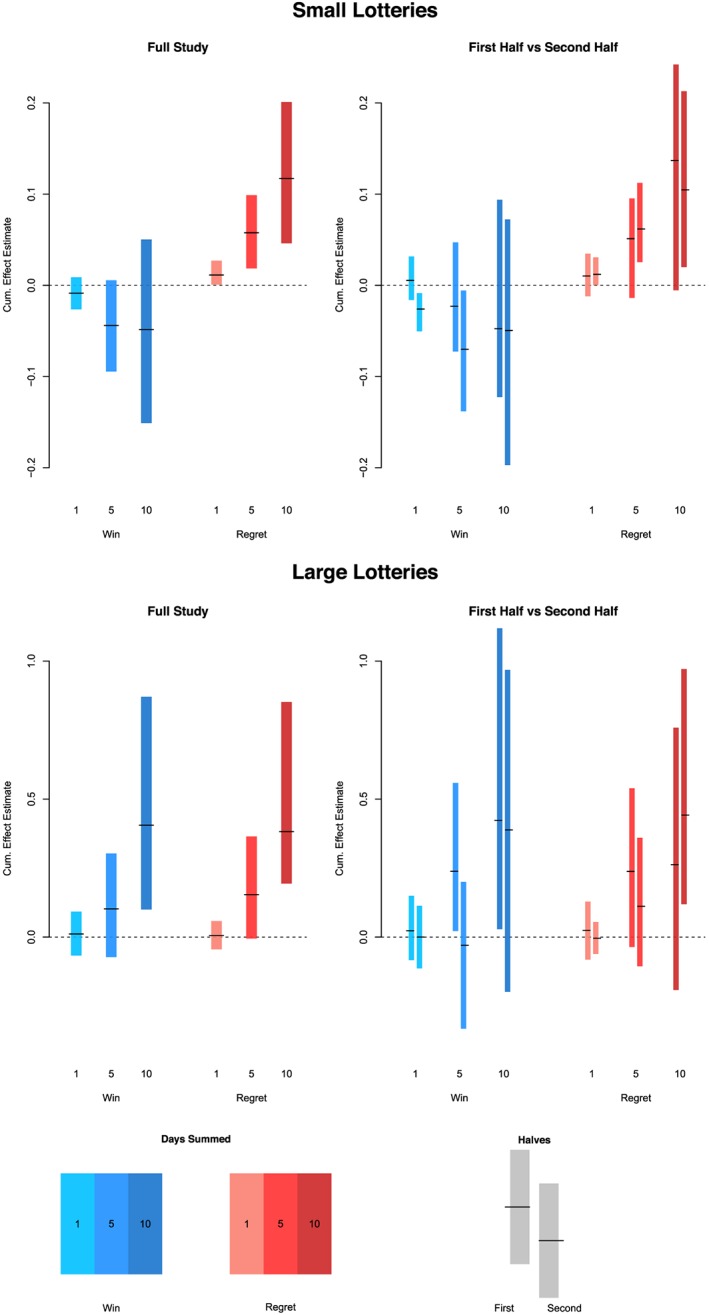
Overall and per‐half estimates of all lottery effects, Shared Incentives. Top left panel gives full study results for small lottery wins and regrets. Top right panel gives results split by first and second half of the study time period. The bottom two plots give results for large lotteries, over the full study (left) and for the two halves (right). Each plot provides win confidence intervals in blue, and regret confidence intervals in red, with the estimate as a black dash, for each of the three short‐term periods considered: 1, 5, and 10 days [Colour figure can be viewed at wileyonlinelibrary.com]

Here, we provide a summary for the Shared Incentives data. The overall and per‐half estimates for small and large lotteries are provided in Figure 2. Blue lines and boxes describe effects for lottery wins; red lines and boxes describe effects for regret messages.

Each colored bar represents a confidence interval around the effect estimate, which is given as the black dash within the bar. 5Note that this is not necessarily in the center of the bar, because these are bootstrapped intervals, not generated from normal approximations. In the plots on the left, we produce estimates and intervals for the full study; in the plots on the right, we split our estimates for each half. Within each plot, the blue bars toward the left display the estimates for wins, and the red bars toward the right display the estimates for regrets. As discussed above, we plot the 1‐, 5‐, and 10‐day estimates for each. Finally, the top panel is for small lotteries, and the bottom panel is for large lotteries.

There is evidence that the small regret messages have a positive and significant effect on adherence, while small wins do not; both large wins and large regret messages appear to have a positive effect of an extra 0.4 adherent days in the 10‐day period after receiving the message. Note that the scales differ for small and large lotteries. There are modest and inconsistent variations in effects between the first and second halves of the study.

We give the total number of matches possible and matches formed in Table 2. We allow duplicated controls in our study. Even with a tight caliper of *δ* = 0.03, we are able to match nearly every lottery result. If we had not allowed duplicated controls, we would have lost over half of our matches, even with just a 3‐day cooling‐off period (slightly less if we had mandated at most one control per treated).

**Table 2:** Total possible matches and total matches formed in the Shared Incentives study. Matches that would have been formed without duplicated controls are provided for context

	Small Wins	Small Regrets	Large Wins	Large Regrets
Occurrences of Lotto type	2825	3507	140	191
# Matches, duplicated controls	2672	3305	132	182
Controls per match	1.93	1.91	1.94	1.91
# Matches, no dup. controls	1091	1374	55	92
Controls per match	1.79	1.81	1.71	1.86

### 5.4 HeartStrong results

**Figure 3:**
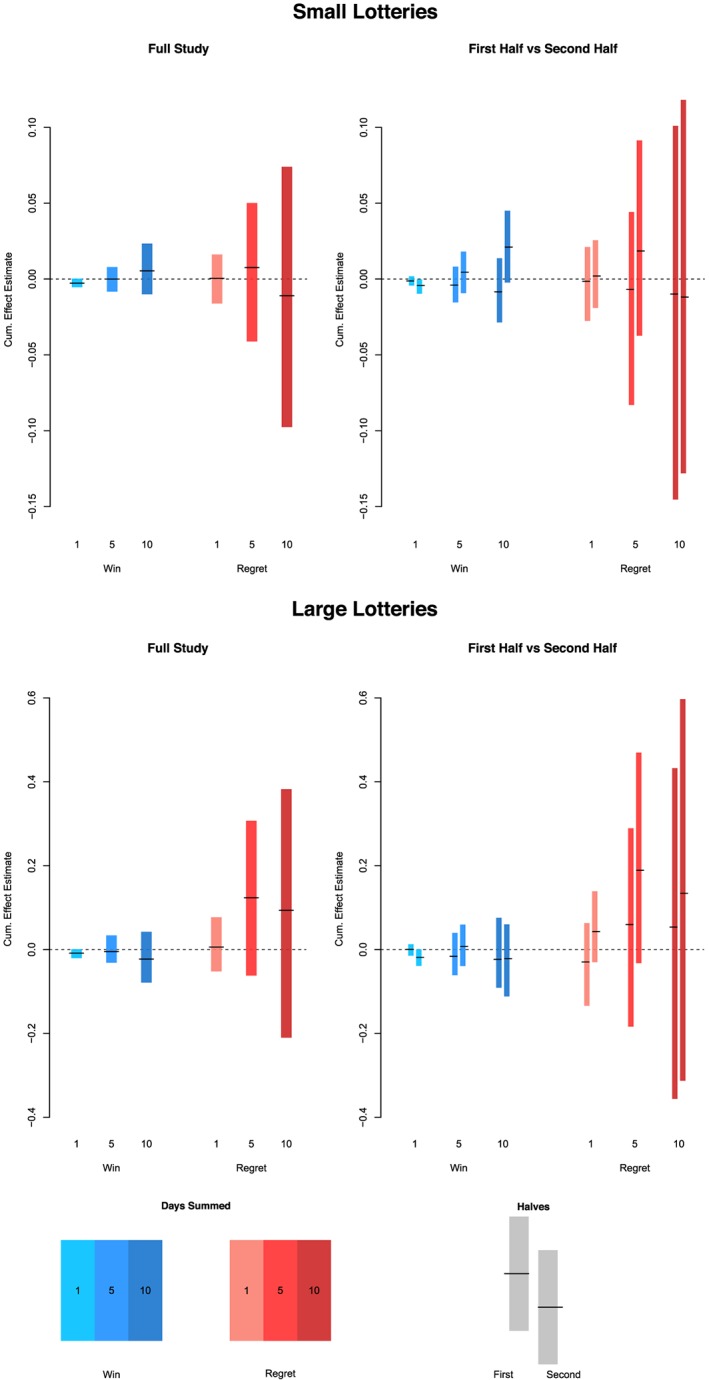
Overall and per‐half estimates of all lottery effects, HeartStrong. Top left panel gives full study results for small lottery wins and regrets. Top right panel gives results split by first and second half of the study time period. The bottom two plots give results for large lotteries, over the full study (left) and for the two halves (right). Each plot provides win confidence intervals in blue, and regret confidence intervals in red, with the estimate as a black dash, for each of the three short‐term periods considered: 1, 5, and 10 days [Colour figure can be viewed at wileyonlinelibrary.com]

We produce the same graphical summaries for the HeartStrong data. The overall and per‐half estimates for small and large lotteries are provided in Figure 3.

Here, we see that the regret estimates have much higher variance than the win estimates, both at the daily level and for longer periods. This is a consequence of very high (92.5%) adherence overall, averaged over participants. Thus, there are far more participants winning lotteries than receiving regret message, both small and large. Overall, there appears to be little to no effect of any lottery type. This is again likely attributable to the very high baseline adherence in this study. There are no discernible differences between the first and second halves of the study.

Similar to the Shared Incentives study, we give the total number of matches possible and matches formed in Table 3. We apply the same matching rules as before, allowing duplicated controls and applying a caliper of *δ* = 0.03. Again, we succeed in matching nearly every lottery result. If we had not allowed duplicated controls, we would have lost approximately two‐thirds of our matches, with just a 3‐day cooling‐off period (slightly less if we had mandated at most one control per treated).

**Table 3:** Total possible matches and total matches formed in the HeartStrong study. Matches that would have been formed without duplicated controls are provided for context

	Small Win	Small Regret	Large Win	Large Regret
Occurrences of Lotto type	33 971	3198	2000	177
# Matches, duplicated controls	33 686	3046	1970	166
Controls per match	1.99	1.88	1.99	1.84
# Matches, no dup. controls	12 500	1046	754	63
Controls per match	1.98	1.61	1.99	1.64

## 6 SIMULATION STUDY

We simulated datasets according to our model, with a factorial design as follows: number of participants (20, 100, or 300); length of sequences per participant (200 or 500 days per person); value of parameters (all zero or randomly nonzero).

Appendix A details some aspects of this simulation. We focus on using the resulting *X* (really Φ(*X*)) values to perform our matching.

For each simulation, we can derive the true value of the lottery at any time point *t* by simulating data up to time *t*, setting the lottery results on day *t* to take our desired value, and then simulating any number of days beyond. This is computed as a function of the parameter means and standard deviations.

For each MCMC result, we calculate the estimated lottery effects as per Section 4.6, and bootstrap the intervals.

**Figure 4:**
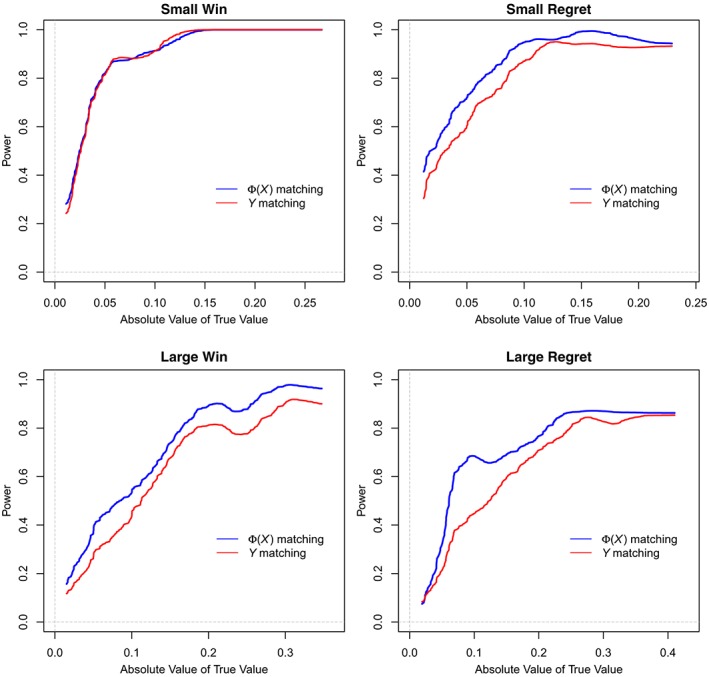
Power as a function of (absolute) counterfactual truth [Colour figure can be viewed at wileyonlinelibrary.com]

Figure 4 plots power as a function of the absolute value of the counterfactual truth. These plots contain two lines. The blue line is for matching as we have described in this paper: run the MCMC, match on Φ(*X*), and compute the estimated lottery effects. The red line is for matching on *Y* itself: at time *t*, compute the running average of the most recent *k* days, and match on those, then compute the estimated lottery effects with these new matches.

For these comparisons, we use *δ* = 0.03, and for *Y*, we match exactly on the mean with *k* = 10, 6Matching exactly on the mean *Y* does not mean we match exactly on the last 10 days, only that two people must have completed the goal the same amount of times in the last 10 days to be matched. which gives approximately the same number of controls for both approaches. We see that Φ(*X*) matching has higher power at all levels of the true value for all lotteries except for small wins, where the two are about equal. We will discuss the method of matching on *Y* further in Section 7.4.

Coverage reaches the nominal value for null lottery effects: we get 95% coverage of zero for simulations with no lottery effect. Coverage drops to 70% in the worst cases at the extreme values of the counterfactual lottery; partly this is due to correlation between the lottery coefficients and the decay coefficients.

We use the simulation study in the following section.

## 7 SENSITIVITY ANALYSES

### 7.1 Length of lottery effect

We somewhat arbitrarily use 1, 5, and 10 days to evaluate the effects of the lotteries. We can use any number of days, although as mentioned in Section 3, we are not trying to assess the total value of the lottery with this analysis, but specifically the shorter‐term effects.

**Figure 5:**
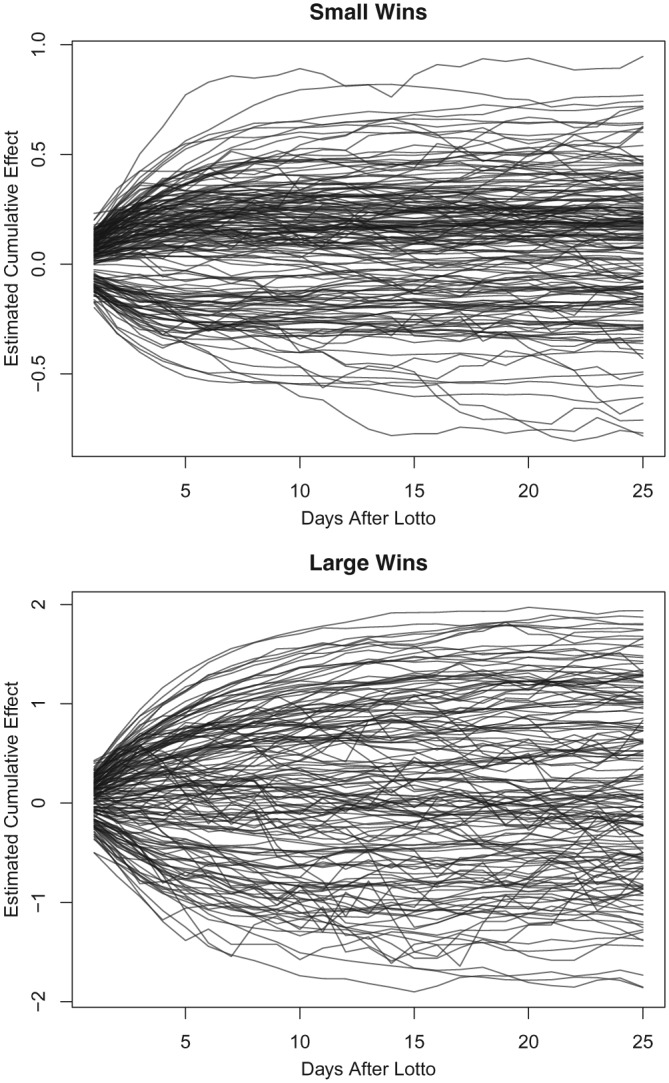
Estimated cumulative lottery effects from one to 25 days

In Figure 5, we plot the estimated effects from our simulated datasets, going to 25 days. In most cases, the extent of the effect is seen after 10 days. In applied work, we can, of course, estimate any number of cumulative days, and the right number will depend on what is of interest to the practitioner. The message of the simulation study is that only very extreme effects would not manifest within ten days.

### 7.2 Control eligibility: matching on propensity

In order for a match to be considered valid, we require a control to be within a given tolerance of a treated subject, as outlined in Section 4.6. If the tolerance is large, we obtain more matches of lower quality. If the tolerance is small, the matches are high‐quality, but the number of matches could be low.

**Figure 6:**
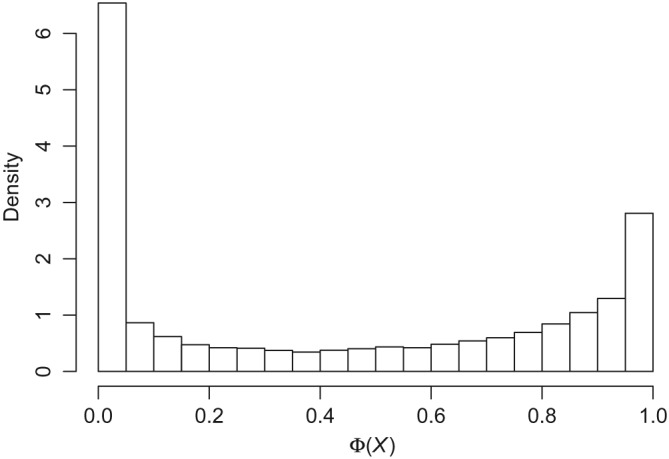
Shared Incentives: histogram of Φ(X)=Pr(Y=1) for all participants and days

In Figure 6, we plot the distribution of the Φ(X) values from the Shared Incentives study as a histogram of all values. We see that there are a large number of participant‐days with very small likelihood of achieving the goal and a moderate number of participant‐days with very high likelihood of achieving the goal, with the remainder with intervening likelihoods. Thus, the availability of controls differs according to the value of Φ(X).

**Figure 7:**
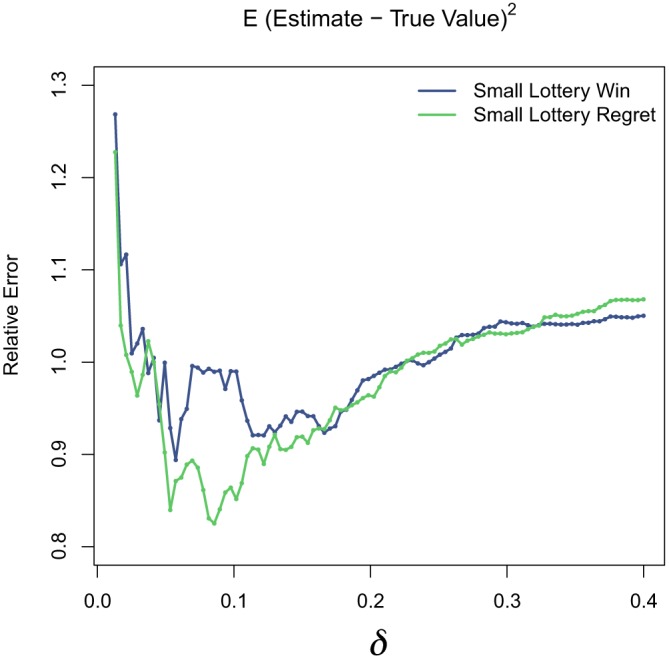
Simulation study: plots of mean squared error for the small lottery, for 1‐day estimates [Colour figure can be viewed at wileyonlinelibrary.com]

Further, in Figure 7, we plot the mean squared errors of the estimates as a function of the tolerance *δ*, averaged over our simulation results. This plot is for the 1‐day effect. When *δ* is very small, the error is high, because many subjects are unmatchable. As *δ* increases somewhat, the error is reduced, reaching its lowest value at around *δ* = 0.01; the error then increases approximately linearly as *δ* increases until *δ* is approximately 0.3. Note that, for these results, we allow at most three controls for each treated subject, so as *δ*→1, we do not tend to allowing random matches, but instead the best three are chosen if all controls are available; this explains why the error remains moderate.

The best *δ* depends on how many participants there are, and to a lesser degree, how many days constitute each series. The small and large lotteries will have different optimal solutions, as will the 1‐, 5‐, and 10‐day effects.

**Figure 8:**
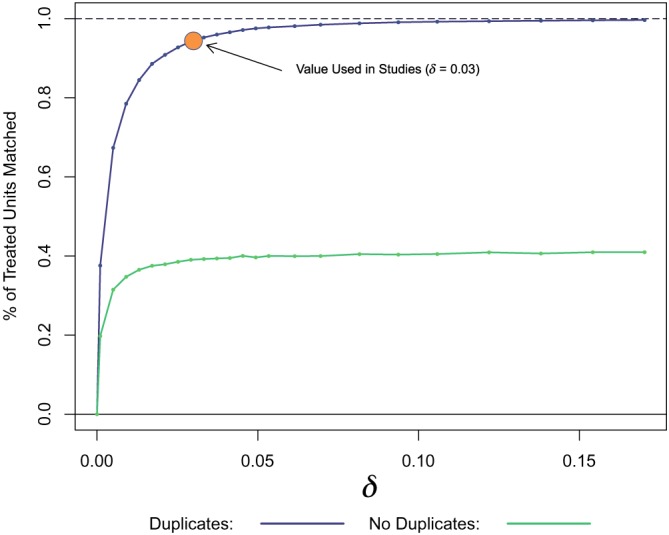
Percentage of treated matches for various calipers, for the Shared Incentives study [Colour figure can be viewed at wileyonlinelibrary.com]

For real data, we cannot assess the bias in the bias‐variance trade‐off separately. Instead, we can plot the number of formed matches as a function of *δ*. This serves as a proxy for variance, especially if we use a bound on the number of controls for each treated subject. Figure 8 plots the percentage of possible matches formed in the Shared Incentives study as a function of *δ*, holding all other variables constant (using the values used for our results: at most two controls per treated). We plot the percentage when duplicates are allowed, and when they are not.

A small *δ* means that some participants on some days might have no valid matches at all, and a large *δ* means that some matches may be essentially random. In our analysis, as in our simulated results above, we restrict to the three closest matches if more than three are eligible. If we were worried about excluding treated subjects who had no match, we could force a match in all cases, even if the two Φ(*X*) values were not close, and only use multiple matches if we had multiple matches available.

Note that our priority is not out‐of‐sample prediction; reducing squared error is not our primary goal. Bias is generally a more significant concern than variance, as bias error is hidden from us. Thus, we tend to favor small values of *δ*. We still care about power, however, so variance is certainly not irrelevant.

### 7.3 Control eligibility: multiple matches

In our matching algorithm, we allow each participant to be involved in multiple comparisons starting on a given study day. We also do not restrict concurrent matching: we allow, eg, a participant to be a control starting on day 11 and then a treated unit starting on day 13.

Avoiding this restricts the number of matches we could create, with the benefit of avoiding correlated differences, eg, if a participant is used as a control from day 11 to day 20, and a treated subject from day 14 to day 23. Depending on the desired *δ* and number of participants, avoiding concurrent matches could have a significant impact on the total number of matches. Here, we evaluate the effect of changing this restriction.

We can increase the variance but potentially decrease the bias by tightening this restriction. We can allow a participant to be freed to be matched after a fixed number of days, eg, five, or as mentioned, extend to a 10‐day restriction, which is equivalent to not allowing a person to be involved in more than one match simultaneously.

Our desire to avoid bias depends on our period of interest. If the 1‐ and 5‐day effects are of primary interest, a 10‐day restriction will prevent matches that might not have caused us concern. However, if the 10‐day effect is of primary interest, we may be more inclined to keep the restriction closer to 10 days, assuming we can justify the restriction with a large sample size.

**Figure 9:**
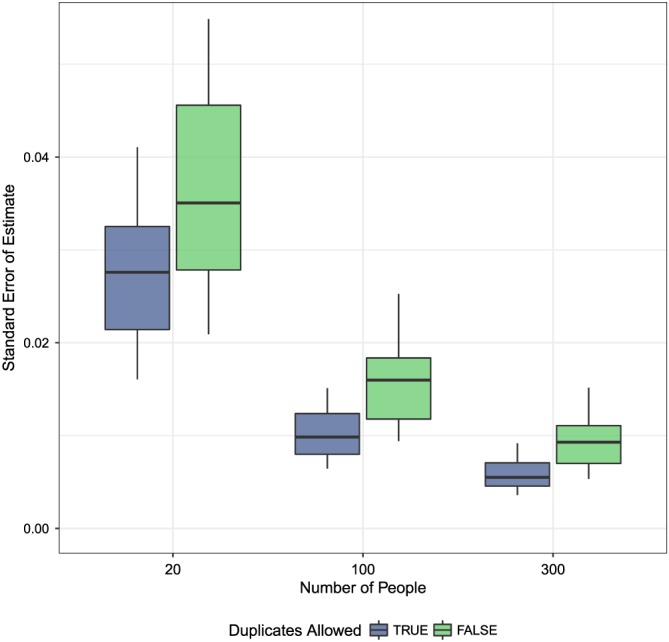
Standard errors of the estimates for small wins, 1 day, for null effects [Colour figure can be viewed at wileyonlinelibrary.com]

Figure 9 plots the standard errors of the estimate for small wins for the 1‐day effect, comparing matching with and without replacement when the true effect is zero. Note that coverage for the duplicated matches is actually slightly higher than the nonduplicated matches. The relationship between the two is the same for nonnull effects.

We can also see the difference in number of matches formed when we allow duplicates, looking at Tables 2 and  3, and Figure 8 from the prior section.

### 7.4 Matching on adherence

Instead of running our MCMC and matching algorithm based on the output, we could instead simply match directly on the ${\left\{Y_{t}\right\}}^{i}$ sequences: for a given day *τ*, we can match participants based on some function of days up to and including day *τ*. For example, we could match on an average of the most recent 10 days, or a weighted average with the most recent days weighted more heavily; we could match exactly on a small sequence of days; we could match both means and number of switches 7A switch is going from adherent to nonadherent on consecutive days, or vice versa. The sequence 010101 has five switches, while 000111 has only one. of the most recent twenty days.

Such a procedure avoids the entire problem of fitting the MCMC. Of course, we may also be interested in the outcome of that model, but for now, we focus on the matching results.

In Section 6, we compared matching on Φ(*X*) to matching on the most recent 10 days; ie, we can match participant 13 and participant 94 on day 17 if

(12) $$\frac{1}{10}\overset{17}{\underset{t=8}{\sum }}Y_{t}^{13}=\frac{1}{10}\overset{17}{\underset{t=8}{\sum }}Y_{t}^{94}.$$

In our simulations, this procedure results in lower power than Φ(*X*) matching (see Figure 4). In our simulations, this was because the variance of the estimates from matching on *Y*, measuring from the counterfactual truth, was higher for all lotteries and cumulative estimates. We do not recommend this procedure as a final outcome, but consider it a useful sanity check on the outcome of the MCMC procedure.

## 8 DISCUSSION

In this paper, we propose a two‐pronged approach to analyzing binary time series with intermediate outcomes. First, we fit a parameter‐driven regression model with a latent process; second, we use the outcome of that model to form matches and estimate effects.

We can view this whole process as an extended propensity match. We first build and fit a latent model: this gives us probabilities for each participant on each day. Secondly, we then use this to form matches, based on minimizing probability distance: this is the same concept behind propensity matching.

Once we form these matches, we break the time series up into segments: a time series interrupted by the lottery results. These matched segments form our lottery estimates, and thus, we have effect estimates on the correct scale.

In our studies, we saw a stronger effect in both cases from the regret messaging. This reinforces what regret theory indicates: we hate to lose more than we love to win.

It is difficult to judge in our studies if the large lottery is sensible, in terms of providing 10 times the effect of the small lottery. In both studies, the large lottery effects do seem larger than the small lottery effects, but the results are consistent with many possible patterns of effect. We could read these results as a validation of having a large and small prize; alternatively, it is possible the large prize dampens the effect of the small one. We also note that it appears that effects in the second half of the study are not overly damped compared to the first half: it is encouraging that participants seem to remain engaged even after 6 months of the program.

A more general question is whether the lottery is sensible at all: the strongest effects measured are 0.5 extra goal completions days in the 10‐day period following the lottery. As discussed in Section 3; however, this “interior” analysis of the lottery is not the best way to answer the question of the lottery's total worth.

## DATA AVAILABILITY STATEMENT

The data that support the findings of this study are available from the corresponding author upon reasonable request.

## APPENDIX A

### SIMULATION STUDY

Each simulation was run with 2000 MCMC iterations; convergence is very fast, from a random start, typically within the first 200 iterations.

Coverage at zero is at the nominal value for all six parameters. Coverage drops to about 80% away from zero without using bootstrapped confidence intervals: bootstrap at the level of participants and run the MCMC algorithm on each bootstrap sample. This can be an expensive step in terms of computation.

The *φ* parameters have one issue near zero, in that the estimate will not be negative. This by itself is not a problem, but the variance of the overall mean is correlated with the *φ* parameter and is worse the closer *φ* is to zero.

The decay parameters are poorly identified, but the log‐likelihood is significantly higher with them than without. The main issue is that the parameters are highly correlated: both the decay and the “length” parameters are correlated, and both are correlated with the respective lottery coefficients. We recommend keeping them for the matching procedure, but we would not trust inference directly on the decay parameters, due to this identification issue.

## APPENDIX B

### HIERARCHICAL STRUCTURE AND GIBBS SAMPLING

For the majority of our parameters, we have a basic hierarchical structure of the form

(B1) $$\beta_{j}∼N(\beta ,\sigma^{2}),$$

where *β* is the parameter of interest, and *β*_*j*_ is the value of that parameter for participant *j*. *β* itself is assumed to come from a very flat distribution with mean zero, and the variance is fit straight from lme4 random effects models. The user can adjust the functions for greater flexibility.

We use this structure on the intercept coefficients, the time coefficients, the small win lottery coefficients, the small regret lottery coefficients, and the *φ* parameters. The exceptions are the large lotteries. Under simulation, we require huge amounts of data to fit individual large lottery parameters to each participant. This is unsurprising, because the average participant only wins a large lottery 3.5 times, let alone a further split into winning and regret. If your data can support individual large coefficients, then, of course, you can fit them.

Note that, from simulations, the lme4 defaults perform very well when compared with pure Bayesian methods, and for the variance parameter, lme4 behaves similarly to flat priors with a spike at zero. lme4 models will also “blow up” when the data cannot support the model, a feature hidden in the form of a bug. This information is what leads us to avoiding fitting individual parameters for the large lotteries.

For the Gibbs sampling, we sample from our generated lme4 models.

#### B.1 Hard to fit data

If the overall adherence is extremely high, as it is in the HeartStrong study, or extremely low, the parameters will not be strongly identified. Similarly, we will have problems if the number of switches in the binary sequences is extremely low.

For the HeartStrong study, this was overcome by having no time coefficient, and only two hierarchical parameters: the intercepts for each participant and the autoregressive parameters. With these restrictions, the MCMC converges very quickly.

## APPENDIX C

### BOOTSTRAPPED INTERVALS AND P‐VALUES

In standard bootstrapping with replacement, we resample our data multiple times, compute our estimates and potentially standard deviations for each resample, and combine those with the estimates on the full sample to produce inferences. Applying this, when we are computing our matched differences, we could sample each treatment‐control combination, potentially many‐to‐one matching, with replacement. But, this would not correctly estimate the variability of our data: if each participant has slightly different lottery behavior, then this method will underestimate the noise of each resample.

Similar to using random effects, we must be slightly more careful when resampling with our data: we should sample *participants* with replacement. This can happen at any stage of inference: we can resample participants when we are calculating our difference estimates, or we could do it from the very start and run the entire MCMC analysis on resampled datasets. Note that this would be expensive.

Generating confidence intervals and p‐values from bootstrap estimates is nontrivial. Our method is the recommended method from the work of Hesterberg^18^; using our bootstrap samples to approximate the distribution of the *t* values produced by differences. This method is from the work of Efron and Tibshirani.^19^

We are interested in some covariate *β*. We use the estimate $\hat{\beta }$, and the standard error ${\hat{se}}_{\beta }$ from our base estimation. In testing against zero, we generate

(C1) $$t=\frac{\hat{\beta }-0}{{\hat{se}}_{\beta }}.$$

This *t* statistic might not have a *t* distribution, due, eg, to lack of independence. We use our bootstrap samples to estimate the distribution instead: we generate *R* bootstrap *t* statistics

(C2) $$t^{*}=\frac{\hat{\beta^{*}}-\hat{\beta }}{{\hat{se}}_{\beta^{*}}}.$$

Setting *q*_*α*_ to be the *α* quantile of the bootstrap *t* distribution, we have

(C3) β^−q1−α2se^β,β^−qα2se^β.

We can reject zero at level *α*. We can further use this to form p‐values by calculating the largest *α* such that this interval contains zero.
